# Propensity or diversity? Investigating how mind wandering influences the incubation effect of creativity

**DOI:** 10.1371/journal.pone.0267187

**Published:** 2022-04-29

**Authors:** Shan-Chuan Teng, Yunn-Wen Lien

**Affiliations:** Department of Psychology, National Taiwan University, Taipei, Taiwan; University of Trieste, ITALY

## Abstract

Mind wandering has been argued to be beneficial for breaking through mental impasses, which leads to better creative performance upon a second attempt (i.e., the incubation effect). However, the evidence is inconsistent. Different from the propensity for mind wandering that has been the focus of past studies, in this study we further examined the role of diversity (i.e., non-repetitiveness of mind wandering respective to its content) and types of mind wandering along the dimensions of intentionality and awareness during incubation when engaging in a 0-back task (a mind wandering-prone condition) and a focused-breathing practice (a mindfulness-induced condition). We proposed that diversity rather than the propensity for mind wandering was crucial for post-incubation divergent creativity and that mindfulness induction would be a more effective way to elicit the incubation effect because it should result in fewer but more diverse mind-wandering incidents than engaging in a mind wandering-prone task. We conducted an experiment with a between-participant variable (incubation tasks: mind wandering-prone, mindfulness-induced, and no incubation). As predicted, the mindfulness-induced group (N = 30) outperformed the control group (N = 31) on flexibility for the unusual uses task measuring divergent thinking after incubation, but the mind wandering-prone group (N = 29) did not outperform the control group. In addition, the diversity of mind wandering and the tendency toward intentional mind wandering predicted the magnitude of incubation effects on flexibility and originality, respectively. Theoretical and practical implications are discussed.

## Introduction

The incubation effect is a phenomenon in which people break through mental impasses after engaging in an unrelated interpolated activity, that is, the incubation period [1, 2]. People with high creative achievement have often cited this phenomenon. One famous example is when French mathematician and philosopher Henri Poincare suddenly came to an idea to connect a mathematic function he had been working on for a while to a remote domain in mathematics while on an unrelated trip that had made him forget about the aforementioned work [3]. Nobel laureate Doherty [4] even suggested that young scholars should engage in familiar and routine activities to elicit creative ideas. What happens during the incubation period has long been regarded as a mystery and remains a topic of interest [1, 2, 5–7]. Some researchers recently have argued that the tendency to mind wandering, occurring when one’s attention shifts to inner thoughts and their consciousness decouples from a target task or the outside world [8, 9], is crucial for incubation [10–13], despite that mind wandering can be detrimental during a creative idea generation phase [14]. Nevertheless, the evidence is mixed. Furthermore, some studies have found that engaging in a mindfulness practice task during incubation, which could reduce mind wandering [15, 16], can be conducive to the incubation effect [17, 18] as well. This further obscures the role of a person’s propensity for mind wandering in eliciting the incubation effect.

We argue that some qualities of mind wandering, rather than the quantity of mind wandering, might be more crucial for the incubation effect. Before going into the details of our proposal, we first review studies pertaining to the issue, particularly the inconsistent findings about the relationship between the incubation effect of creativity and the tendencies for mind wandering and mindfulness. Then, we present our hypothesis and the rationale for an experiment we conducted to test the hypothesis.

### Tendency to mind wandering and the incubation effect

Although many have long speculated that some type of associative process during the incubation period is related to breaking through mental impasses afterward [5, 7, 19–21], the potential facilitating role of mind wandering in the incubation effect was not a topic of concern in psychology until Sio and Ormerod’s [2] meta-analysis study. They found that engaging in a cognitively undemanding task (i.e., a low-load task) was generally more likely to elicit the incubation effect than engaging either in a demanding task or in resting. Because people’s minds typically wander more during low-load tasks compared to during high-load tasks [22–25], it is not surprising that a corollary hypothesis assumes that mind wandering assists in breaking through mental impasses, which we will call “the mind wandering account.” However, empirical evidence about whether mind wandering benefits the incubation effect remains inconsistent.

Baird et al. [10] first reported supporting evidence for this claim. The researchers found that participants engaging in a low-load task (i.e., the 0-back task) performed better on the originality scores of the unusual uses task (UUT), which is a widely used measurement of divergent thinking, upon a second attempt compared to those engaging in a high-load task (i.e., the 1-back task), resting, or solving problems twice in a row [10]. Consistently, the low-load group retrospectively rated their tendency toward mind wandering during incubation higher than the high-load group did, although comparably with the resting group. Based on these findings, Baird et al. [10] claimed that more mind wandering led to higher chances of generating different ideas through unconscious associative processes. Note that they did not directly examine whether the propensity for mind wandering during incubation correlated with the magnitude of the incubation effect, which would be a more direct support for their account. It is also worth noting that the low-load group did not report a higher tendency for mind wandering than the resting group did, but the former still outperformed the latter for the improvement upon the post-incubation. Zedelius and Schooler [13] suggested that compared with the thoughts or daydreams generated during rest, those generated when engaging in a low-load task might be more varied and activate wider memory representations, which has not yet been examined.

A following finding that Yamaoka and Yukawa [26] reported was in line with the mind wandering account to some extent. They adopted the experiment design identical to Baird et al. [10] except they adopted a more demanding high-load task (i.e., the 2-back task). The researchers found that participants who retrospectively rated themselves high in mind wandering tendency during incubation outperformed those who rated low for the improvement in the originality and flexibility scores of the UUT upon the post-incubation when participants were collapsed across the resting, low-load, and high-load groups. However, unlike Baird et al. [10], they did not find any group difference in the incubation effect. Recently, Yamaoka and Yukawa [27] replicated the findings regarding UUT flexibility scores with different interpolated tasks (i.e., continuing idea generation but not writing down, engaging in a high-demanding Sudoku task, and taking a rest).

Unlike the previous studies, Smeekens and Kane [28] measured mind wandering during incubation with the online probe-caught method and examined the correlation between the probe-caught mind wandering and improvement in UUT performance upon a second attempt. They conducted three experiments that adopted a low-load task resembling that of Baird et al. [10] (i.e., the 0-back task), a high-load task (i.e., the 2-back task), and a reading task as the interpolated tasks respectively. They found no significant correlation between mind wandering and UUT performance in any of the experiments. Note that they did not use a control condition to gauge any potential incubation effect.

Steindorf et al. [29] argued that online thought probing used in Smeekens and Kane [28] might disrupt the associative dynamics of mind wandering, which can be crucial for creative incubation, and thus resulted in the null results. To examine their conjecture, they compared the UUT’s incubation effects in three incubation conditions: a low-load task with thought probes, a low-load task with trivia probes (in which participants responded to a random knowledge question), and a low-load task without probe. They conducted a retrospective assessment of mind wandering after the incubation period for all conditions. A group who solved problems twice continuously without any distracting task was used as a comparison group. They found no group difference for the incubation effect or the retrospective ratings of mind wandering, indicating that the lack of a beneficial effect of mind wandering on incubation in Smeekens and Kane’s study [28] could not simply be attributed to additionally using thought probing. Again, no significant correlation was found between the tendency to mind wandering (online probed or retrospectively assessed) and the second attempt performance for any incubation group, which replicated Smeekens and Kane’s findings [28].

Recently, Murray and colleagues [30] attempted to replicate Baird et al.’s [10] findings with a larger sample. However, no difference in the improvement of UUT performance upon a second attempt was found between the low- and high-load groups, even though the former had more probe-caught mind wandering than the latter. In addition, no correlation between probe-caught mind wandering and improvement in UUT performance was found for any group, which was also consistent with Smeekens and Kane’s [28] findings.

Regarding convergent creativity, the mind wandering account is partially supported. Tan et al. [11] reported that the tendency toward mind wandering measured with online probes during incubation was higher for those who succeeded in a rule-discovery task during the second attempt compared to those who failed, given their working memory capacities were comparable. However, the successful group also rated themselves as more creative compared with those who failed, which was a potential confounder for the positive result. Another study using the compound remote associates task further indicated that the mind wandering account was supported only when previously unsolved problem items were repeatedly presented as part of the stimuli in the interpolated task [12]. However, this result could be explained alternatively. That is, participants with more probe-caught mind wandering could have spent more time on the presented unsolved problem items while their minds were wandering than those with less mind wandering did.

In sum, although the mind wandering account of the incubation effect is intuitively appealing, the evidence remains small and inconclusive. Only half of six studies on divergent thinking found supporting evidence, and this inconsistency cannot be attributed to difference in mind wandering assessment. In studies on convergent thinking, the mind wandering account only stood under some conditions and had to compete against alternative explanations.

### Two subtypes of mind wandering and the incubation effect

To resolve the above inconsistent findings on the relationship between mind wandering and the incubation effect, some researchers have argued that whether mind wandering was beneficial for eliciting the incubation effect might depend on its type [28, 30, 31]. Researchers have often examined two subtypes of mind wandering respect to various types of cognitive performance, including creativity. The intentionality dimension of mind wandering refers to whether mind wandering occurs intentionally [32, 33]; whereas the awareness dimension refers to whether an individual is aware of their disengagement from the current task [34, 35].

Some have suggested that creative people tend to engage intentionally in mind wandering when at rest or doing undemanding tasks [13, 28, 36]. Zedelius and Schooler [13] further suggested that intentional mind wandering could open an opportunity to inspire creative ideas about an unfilled goal or unsolved problem. Agnoli et al. [37] further reported the first piece of empirical evidence showing that only the self-rated tendency toward intentional mind wandering in daily life positively correlated with originality scores on the UUT. They suggested that having control over the mind-wandering state might be conducive to introducing apparently unrelated thoughts to the creative focus into thinking process, leading to increased originality. However, whether online intentional mind wandering during the incubation period could also facilitate the creative performance afterwards remains unknown. We thus examine it for the first time.

Regarding the awareness dimension, Schooler found that participants who were aware of their mind wandering also performed better on UUT (an unpublished finding cited in Glausiusz [38]). In contrast, Zedelius et al. [39] found that the self-rated tendency toward unaware mind wandering in daily life was positively correlated with the tendency toward mind wandering with fantastic content, which is often related with creative ideas. However, they did not find a significant correlation between the trait-like tendency toward unaware mind wandering and divergent or convergent creative performance. Tan et al. [11] first examined whether the tendency toward aware mind wandering during incubation facilitated creative performance afterwards. However, they found that the degree of awareness of probe-caught mind wandering was not correlated with the incubation effect pertaining to a rule-discovery task that involved more convergent rather than divergent thinking processes. To our knowledge, none have examined whether awareness of mind wandering plays a role in eliciting the incubation effect regarding divergent creativity. Here, we explore this topic.

### Mindfulness and the incubation effect

As mentioned above, the findings that mindfulness practices benefit the incubation process further challenge the mind wandering account. Mindfulness, a psychological construct originating from Buddhism and associated contemplative practices, usually refers to a state or trait-like tendency to be aware of and attentive to the present moment nonjudgmentally [40–42]. Past studies have shown that mindfulness or meditation induction and practices decrease one’s tendency to mind wandering afterwards [15, 16, 43–46]. Some studies also show that an individual’s tendency to mind wandering decreases during mindfulness induction compared to focusing on a mental image [47] or resting [48]. Participants with high trait mindfulness also showed less mind wandering during undemanding tasks [16, 25, 47, 49–51]. Thus, mindfulness was often considered as the opposite construct to mind wandering in terms of the ability to remain undistracted [16].

However, in reality, mind wandering and mindfulness states coexist during any mindfulness or meditation practice. Although instructions for being mindful or meditative vary, a common key mindfulness or meditation practice is to cease or reduce the elaborative thinking process. This can be achieved through being fully concentrated on and aware of one’s own breaths, bodily sensations, a mantra, or just passively and objectless waiting for the sensations (or thoughts) to come and go as a nonattached witness (e.g., [40, 42, 52–57]). However, individuals can only sustain such a “quiet” or mindful state intermittently because mind wandering, such as thinking about one’s current concerns, returns from time to time. For example, Hasenkamp et al. [58] showed that even expert meditators reported mind wandering every 80 seconds, on average, over a 20-min meditation session, not to mention the beginners.

In addition, researchers have found some aspects of trait mindfulness and mind wandering to work interactively on creativity. Agnoli et al. [37] reported that people who were aware of their present inner or outer experience, which is a main characteristic of trait mindfulness, had higher originality scores on the UUT if they also reported a higher tendency to intentional mind wandering. Therefore, the researchers called for studies adopting experimental approaches to investigate the possibly complex interaction of mind wandering and mindfulness in various forms of creativity.

Thus, in the current study, we included a mindfulness-interpolated task and examined its potentially beneficial effect on incubation from a perspective of mind wandering. Before discussing our proposal and predictions, we first review literature regarding the effect of mindfulness induction on incubation. To our knowledge, only three studies have investigated that, although numerous studies have revealed that engaging in various types of mindfulness inductions or meditation practices could enhance performance on both divergent and convergent thinking tasks [59–63].

Ren et al. [18] first discovered that participants engaging in breath-counting meditation (a focused-attention type of mindfulness practice) during incubation exhibited greater improvements in convergent thinking when assessed with insight problems compared to those engaging in a high-load task. Kudesia et al. [17] explored two conditions, practicing an open-monitoring type of mindfulness skill to be in a mindful state and engaging in a low-load cognitive task to be in a mind wandering-prone state during incubation. They revealed that participants from the two conditions together generated more ideas that were new during the second UUT attempt compared to those engaging in a high-load task. Furthermore, participants engaging in mindfulness practice generated more far-reaching ideas (as flexibility on the UUT and performance of insight problems indicated) than combined low- and high-load groups did. The researchers suggested that mind wandering engaged associative processes that only led to ideas most strongly associated with the initial ideas, while openly “monitoring” one’s thoughts in a mindful way could enhance creativity through avoiding immersion into habitual or potent thoughts, which often suppress conceptually dissimilar ideas. Note that Kudesia et al. [17] neither directly compared the incubation effects that the mindfulness practice and the mind wandering-prone task brought up nor assessed mind wandering during incubation, leaving the “free from potent thought” explanation unexamined.

Recently, Rummel et al. [64] assessed retrospectively the tendency to mind wandering while performing three interpolated tasks during incubation, including a mindful body-scan practice, a low-load task, and a high-load task. As expected, the mindfulness group generated less mind wandering than the other two groups did. However, no group difference in the post-incubation performances (solving magic tricks) was found. This result, again, did not support the mind wandering account or the effect of mindfulness practice on incubation. Note that unlike the two previously mentioned studies, participants in Rummel et al. [64] only saw but not actually solved creative problems before the incubation period, so that none of them could be considered encountering any mental impasse before the incubation period.

### Our proposal: The diversity rather than the propensity of mind wandering matters

Unlike the mind wandering account, we propose that an often-ignored aspect regarding non-repetitiveness of mind wandering with respect to its theme, which we call the “diversity” dimension, might be more crucial compared to the propensity dimension for eliciting an incubation effect on divergent thinking. Here, we define the high diversity of mind wandering as having no or few dominant or potent ideas that occupy the off-task thoughts and thus is more likely to have dissimilar or distant thoughts, but not necessarily higher propensity of mind wandering. In other words, the more that mind-wandering incidents linger on the same theme, the lower the diversity of mind wandering is.

Our proposal is based on two different lines of research regarding mind wandering and mindfulness, both of which imply a potential link between diversity of mind wandering and creativity, respectively. The first one is related to the unconstrained feature of mind wandering emphasized in the dynamic framework of mind wandering proposed by Christoff and colleagues [65–67]. In contrast to the task-centric view of mind wandering, which considers mind wandering as task-unrelated thoughts and thus focuses on the propensity to be off-task, these researchers regarded mind wandering (or daydreaming) as a cognitive state involving an unconstrained or unguided process. In other words, mind wandering is a kind of spontaneous thought that arises relatively freely without strong constraints on contents or topical focuses, which lies in contrast to producing thoughts with repetitive contents or restricted topics (i.e., rumination and goal-directed thoughts) [65, 68]. Thus, it implies that the free-moving process would generate variable thoughts not tied to some potent thoughts, as the two sides of a coin. Indeed, Christoff et al. [65] have explicitly expressed that “thought becomes spontaneous and more variable when deliberate and automatic constraints are relaxed.” This is similar to the diversity dimension of mind wandering we define. Furthermore, this free-moving cognitive process can activate different mental representations or remote associations and benefit the process of creative idea generation [65, 68]. We thus reason that the diversity of mind wandering during incubation is a plausible candidate to elicit the incubation effect regarding divergent thinking, which emphasizes generating new ideas.

The second line of research, on which our proposal is based, is related to how mindfulness practices or induction could reduce repetitive thoughts. Particularly, the diversity aspect of mind wandering is relevant to the “free from the potent thought” explanation of the incubation effect regarding the mindfulness induction we mentioned previously [17], and is used to examine this explanation in a more direct way in the current study. When noticing an incident of mind wandering during a mindfulness or meditation practice, meditators will bring back their attention to the breathing process or bodily sensations and cease possible ongoing elaborative thinking again. This is supposed to prevent an individual from immersing into potent thoughts or associated themes (e.g., [42, 53, 69]) and give a chance to redistribute activation away from their initial thought [17], which is likely to increase variability of thoughts. Thus, the “free from potent thought” explanation Kudesia et al. [17] proposed also implies that diversity of mind wandering would increase while doing mindfulness practice. In addition, accumulated evidence shows that cognitive flexibility and discontinuation of automatic cognitive processing could be enhanced, indicated by the reduction of ruminative thoughts and obsessive thinking, after a short-term mindfulness practice or a brief induction [70–75] (for a review, see [76]). Moreover, it was found that engaging in a brief mindfulness induction not only reduced the propensity of mind wandering, but also reduced repeated intrusion of an unwanted thought (in some sense, increasing the diversity of mine wandering) during the induction [47].

Therefore, through different routes, we suggest that engaging in both a mind wandering-prone task and a mindfulness-induced task can possibly increase the diversity of mind wandering. On one hand, mind-wandering proponents implicitly assume that generating more mind wandering leads to a higher chance of eliciting more varied thoughts through unconstrained or unconscious associative processing [10, 26, 30, 77]. Indeed, engaging in an undemanding task was recently found to involve a more unconstrained process than engaging in a high-load one [78]. On the other hand, as elucidated above, engaging in a mindfulness-induced task could increase the diversity of mind wandering via the avoidance of immersing into potent thoughts. With the new perspective of diversity, these seemingly contradictory findings about the incubation effect of mind wandering and mindfulness, if the propensity of mind wandering is focused, could thus be reconciled and incorporated.

Nevertheless, we predicted that engaging in mindfulness induction could be a more reliable way to increase the diversity or non-repetitiveness of mind wandering compared to engaging in a mind wandering-prone task. This is because current concerns or worries are likely to be repeatedly elicited or spontaneously arise as an individual’s mind wanders [31, 79, 80], which involves the constrained goal-directed or automatic process. The degree of diversity of mind wandering a low-load task brings up thus depends on the strength of current concerns or worries that an individual holds. Again, this indicates that the high propensity for mind wandering does not necessarily lead to a high diversity of mind wandering.

In sum, we hypothesized that the diversity of mind wandering, not the propensity for mind wandering during incubation, should predict the magnitude of the incubation effect, particularly for far-reaching ideas in divergent thinking tasks (i.e., the flexibility scores) [17]. Furthermore, engaging in mindfulness induction during the incubation period could generally produce, on average, fewer but more diverse mind wandering and thus better elicit the incubation effect compared to engaging in a mind wandering-prone task.

### The current study: Rationale and predictions

To test our hypothesis, we compared the incubation effects of two types of incubation tasks resulting in two different states of consciousness—a mind wandering-prone state and a mindfulness-induced state. We measured three dimensions of mind wandering—diversity, intentionality, and awareness—in addition to participants’ propensity for mind wandering while engaging in these tasks. In addition, we measured individual differences in trait mindfulness, which was negatively correlated with the propensity for mind wandering [16, 25, 47, 49–51], to contorl the baseline mind-wandering tendency across groups.

We adopted a between-participants design with an incubation paradigm. We used two interpolated tasks during the incubation period: a 0-back task resembling previous studies [10, 28–30] in the mind wandering-prone condition, and a focused-breathing task in the mindfulness-induced condition. In contrast to Kudesia et al. [17], who used the open-monitoring method to induce a mindfulness state, we adopted a skill emphasizing breathing with an empty mind derived from the East Asian tradition [47, 52]. This skill involves an individual monitoring their breaths and the associated body sensations with their eyes relaxed, looking both downwards and inwards. In open monitoring, participants are usually instructed to monitor or observe their thoughts, sensations, and feelings, letting them freely arise and pass through their minds without clinging to them [69, 81, 82]. The open-monitoring practice decreases identification with thoughts and mood states, thereby reducing mind wandering. However, without first practicing focused-attention skills to enhance attentional stability, novices may generate many thoughts when practicing open monitoring [82]. In other words, practicing such a skill in a short time could enhance a beginner’s diversity of mind wandering as well as their propensity for mind wandering, making it difficult to attribute the observed effect, if any, to a particular factor. To better decouple diversity from the propensity of mind wandering, we thus chose a focused-breathing type of meditative skill, which has been demonstrated as an effective means for reducing mind wandering in a short time for novices [47].

We sampled for the tendency toward mind wandering in general and for the subtypes along the dimensions of intentionality and awareness, in particular using online thought probes while performing the interpolated tasks. We chose this method because of the difficulty in distinguishing subtypes of mind wandering retrospectively. In addition, it does not rely on participants’ ability to be aware of or memorize conscious states [83, 84]. Instead, the participants judged the diversity of mind wandering immediately following the incubation period because reviewing one’s stream of consciousness throughout the whole period was needed for this judgment.

In addition, to reduce possible disturbances that thought probes caused, the intervals of adjacent probes were set to at least 60 seconds for both incubation conditions (100 seconds on average). The intervals of adjacent probes were longer than those that Smeekens and Kane [28] (48 seconds on average), Steindorf et al. [64] (56 seconds each), and Murray et al. [30] (64 seconds on average) used. Because past studies have found a positive correlation between the interval of adjacent probes and the tendency to mind wandering [85, 86], our design made participants in the mind wandering-prone condition go off-task more easily than in the previous studies.

We measured the magnitude of the incubation effect in terms of improvement in the three UUT indices after incubation. As a divergent thinking task, we selected the UUT because it can produce the incubation effect in a test environment [2]. It also involves an idea generation process, which is likely to be associated with diverse mind wandering as mentioned, compared to the evaluation process of creativity.

As for the index of improving UUT, most of the aforementioned studies adopted the percentage improvement, which was calculated as [(UUT score of the second attempt–UUT score of the first attempt)/(UUT score of the first attempt)] × 100 [10, 28–30]. Although this index seemed reasonable, it might result in a potential confounding, leading to the inconsistent findings. To be specific, such calculation did not exclude the ideas that had been generated on the first attempt when measuring the improvement in creativity on the second attempt. Thus, some improvement found on UUT might be due to the participants’ better retrieval of old ideas generated on the first attempt rather than generating more ideas that are new after incubation. Consequently, this index might not precisely reflect the true improvement of UUT. Therefore, in the present study, we calculated the improvement of UUT only based on the newly generated ideas on the second attempt, similar to as Kudesia et al. [17] and Yamaoka and Yukawa [26] did.

Finally, researchers have argued that incubation should be especially effective after individuals become stuck on solving problems or generating creative ideas, and that mind wandering likely facilitates creative performance particularly when a mental impasse has been reached [13, 87]. It could be the case that, for those who did not become stuck during the first trial, the improvement in a second trial might just be due to their superiority at generating creative ideas. However, whether participants encountered impasses has never been considered in extant studies on the incubation effect regarding divergent creativity and this could be a possible confounding variable. To better scrutinize the incubation effect, the present study assessed whether participants encountered mental impasses during the first attempt. Participants who did not encounter mental impasses were excluded from further analyses.

We predicted that the mindfulness-induced group would have fewer but more diverse mind wandering during incubation compared to the mind wandering-prone group, and thus would show a better incubation effect on UUT, particularly for the index of flexibility. Furthermore, we expected the diversity, but not propensity of mind wandering, would predict the magnitude of improvement upon the second attempt of UUT. We also explored the predictive role of intentional/unintentional and aware/unaware mind wandering in the improvement of UUT performance. Particularly, a potential positive link between the tendency of intentional mind wandering during incubation and the improvement in originality was expected according to Agnoli et al.’s [37] findings.

## Methods

### Participants

In total, 108 undergraduate and graduate students from National Taiwan University (64 female students; mean age = 20.96 years old, SD = 1.71 years) were recruited through the internet and paid NT$200 (approximately USD $6.50) for their participation. We calculated the sample size needed to detect at least a medium-sized effect Baird et al. [10] reported (*ƞ*^*2*^ = 0.10) with a power greater than .80 (two-tailed), for a between-participants design. A sample size equal to or larger than 90 was suggested using G*Power software [88]. The study was approved by the research ethics committee of National Taiwan University. Participants provided written consent prior to the commencement of the study.

### Design

We conducted an experiment with a between-participants design and an incubation paradigm. All participants were randomly assigned to one of the following three conditions: (a) the mind wandering-prone (MW-prone) condition, in which participants engaged in a low-load cognitive task (the 0-back task) during the incubation period between the two UUT attempts; (b) the mindfulness-induced (MF-induced) condition, in which participants engaged in a focused-breathing task during the incubation period; and (c) the control condition, in which participants performed the UUT twice consecutively without engaging in a distracting task between the two attempts.

The dependent variables were the three creativity indices of the UUT—fluency, flexibility, and originality. For the two incubation groups, we measured four aspects of mind wandering during the incubation period—propensity, diversity, intentionality, and awareness. Furthermore, we measured an individual difference factor, mindfulness, for all participants.

### General procedures

**Fig 1:**
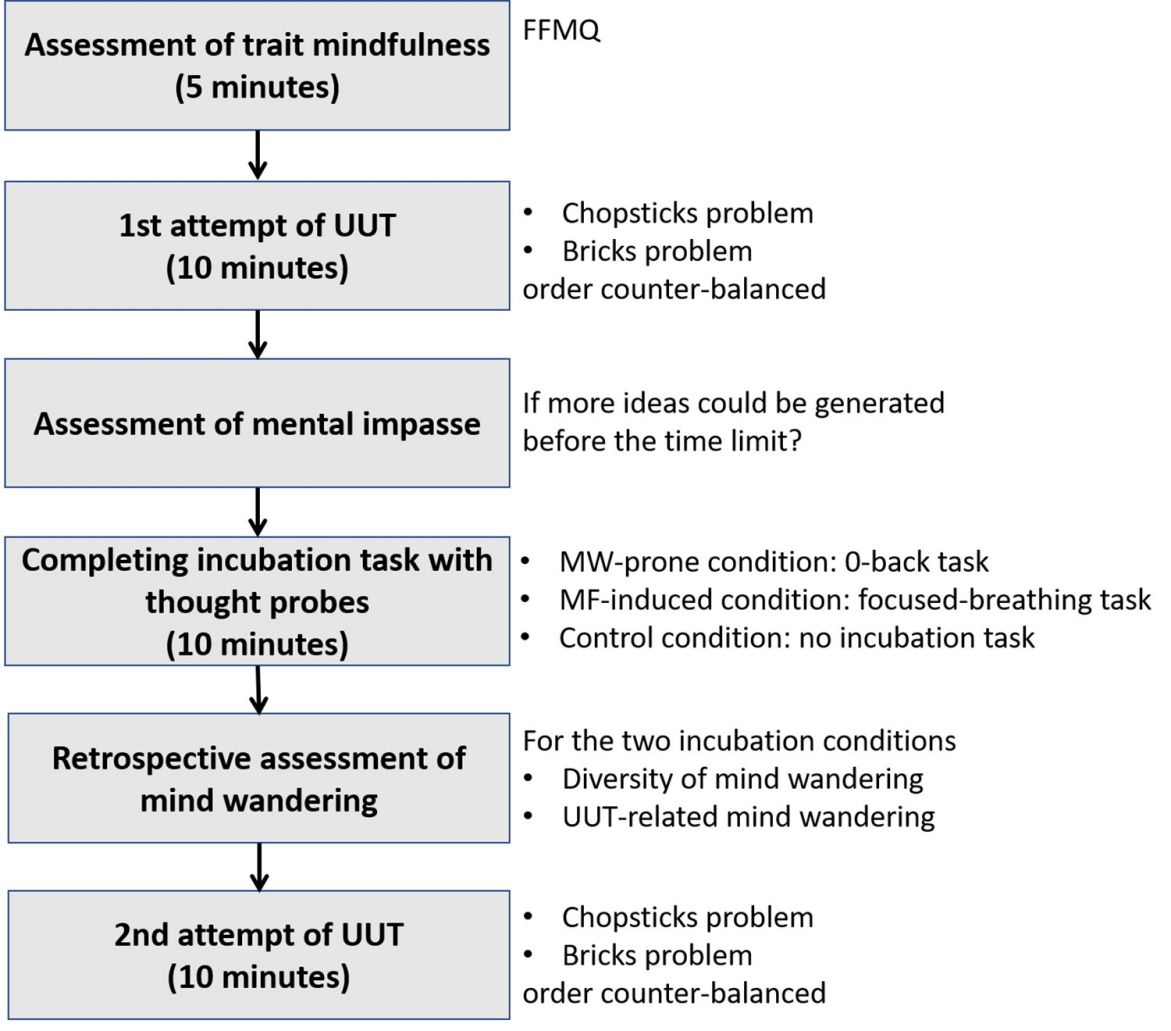
The general procedures of the experiment.

The general procedures are depicted in Fig 1. The participants were individually tested in a quiet room. First, the participants were asked to complete the Five Facet Mindfulness Questionnaire (FFMQ) to measure mindfulness. Then, they worked on two UUT problems (the chopsticks and bricks problems, 5 minutes each) one by one. The presentation order of the problems was counterbalanced among participants.

At the end of each problem, we asked participants whether they had encountered a mental impasse (i.e., no more ideas could be generated before the time limit). The participants then performed the second attempt without being notified beforehand.

For the two incubation conditions, the participants performed an interpolated task between the two UUT sessions for 10 minutes. We employed a thought probe method during the incubation session to detect possible mind wandering while the participants were engaged in the interpolated tasks. Upon completing the interpolated tasks, the participants were asked to rate (a) how often they thought about the UUT problems during the task on a scale from 1 (never) to 10 (always), and (b) how diverse their mind wandering was on a scale from 1 (only had one thought during mind wandering) to 10 (never had the same thought more than once during mind wandering). The control group solved each problem twice consecutively without an incubation period.

### Materials and tasks

#### UUT

We used two problems from a Chinese version of the UUT [89]. We instructed participants to type as many unusual uses for an everyday object (bricks or bamboo chopsticks) as they could in 5 minutes. Three indices of divergent creativity were measured for each participant: (a) fluency, the number of uses that a participant reported; (b) flexibility, the number of different categories to which a participant’s responses belonged; and (c) originality, the number of infrequent uses reported (i.e., those that less than 1% of participants generated). The participants’ raw scores along each index were first transformed into z-scores for each problem. The final score of each index was averaged over the two problems.

#### Thought probing and the low-load task

A 0-back task was used as the interpolated task in the MW-prone condition. It has been demonstrated that mind wandering likely occurs during this cognitively undemanding task [25]. In this task, a series of colored digits were presented, one at a time, in the center of a screen. The participants had to judge whether the digits were odd or even by pressing the corresponding keys as quickly as possible if the digit was red but not if the digit was white.

The task consisted of six blocks of trials. Each block included 24, 30, 35, 44, 49, or 58 trials for a total of 240 trials. In each trial, a single-digit number (1–9), colored either red or white, was presented for 0.5 seconds against a black background, and followed by a blank screen for 2.0 seconds. In each block, one-third of the digits were red (i.e., the target trials), and the remaining digits were white (i.e., the nontarget trials). The sequences of the six blocks were randomly determined for each participant.

In addition, six thought probes appeared on the screen at the end of each block. The participants had to choose an option according to whether they had been concentrating on the task the moment before the probe appeared. The participants were then asked to answer two additional questions by pressing a designated key if their minds were not engaged in the task: (a) “Were you aware of what you were doing just then?” and (b) “Did you intend to do that?” Based on their responses, the caught mind wandering was classified as aware or unaware and intentional or unintentional, respectively. The number of probe-caught mind-wandering incidents, which ranged from 0 to 6, indicated the tendency toward mind wandering. The proportion of probe-caught aware/intentional mind wandering out of all probe-caught mind-wandering incidents, which ranged from 0 to 1, indicated the likelihood of aware and intentional mind wandering.

The participants performed a practice block of 21 trials before the formal trials commenced. The participants had to achieve an accuracy rate of 70% on the practice trials before proceeding to the formal trials.

#### Thought probing and focused-breathing task

For this task, the participants were instructed to concentrate on their breathing for 10 minutes as follows [47]:

*Please close your eyes and sit straight, but in a way where you feel relaxed. Concentrate on your breaths. Breathe in and out slowly and deeply. Feel the air coming through your nose, filling up your chest and belly. Let your eyes go inward and downward while exhaling. Every time you notice your attention has shifted away from your breath, take a deep breath, redirect your attention to your breathing, and keep your eyes looking inward and downward when exhaling*.

Similarly, six thought probes were presented, along with a “ding” sound during the task at intervals of 60, 74.4, 86.4, 110.4, 122.4, and 146.4 seconds. The intervals approximately matched the length of each block in the 0-back task. The presentation order of the time intervals was randomly determined for each participant, which was also done for the 0-back task. For each probe, the participants had to answer whether they had been focusing on their breaths without thinking of anything else just before the “ding” sound by pressing a designated key while their eyes were closed. If their minds had wandered, the participants were asked two additional questions identical to those in the 0-back task to classify their mind wandering in terms of awareness and intentionality. Again, the number of times the participants did not focus on their breathing indicated a tendency toward mind wandering, and the proportion of probe-caught aware/intentional mind wandering out of all probe-caught mind-wandering incidents indicated the likelihood of aware and intentional mind wandering. The participants performed two 2-minute practice sessions before the formal trials.

#### FFMQ

A Chinese version of the FFMQ [90], translated and revised from Baer et al. [91], was adopted to measure the participants’ trait mindfulness. The FFMQ evaluates five facets of mindfulness, with Cronbach’s α = 0.79–0.92: (a) the nonreactivity of inner experience, or allowing feelings and thoughts to come and go without losing attention (e.g., “I watch my feelings without getting lost in them”); (b) observing or attending to internal or external stimuli (e.g., “I notice the smells and aromas of things”); (c) acting with awareness or attending to one’s current experience (e.g., “I don’t pay attention to what I’m doing because I’m daydreaming, worrying, or otherwise distracted” [a reversed item]); (d) describing, noting, or mentally labeling experienced stimuli with words (e.g., “I’m good at finding the words to describe my feelings”); and (e) the nonjudgment of inner experience, or abstaining from judging sensations, cognitions, and emotions (e.g., “I think some of my emotions are bad or inappropriate and I shouldn’t feel them” [a reversed item]).

The participants rated these items according to their daily experiences on a 5-point Likert-type scale ranging from 1 (never or very rarely true) to 5 (very often or always true). The average score for all facets indicated the participants’ trait mindfulness. Higher scores indicated a higher degree of mindfulness in general.

## Results

Based on their reports, 10 participants who did not encounter mental impasses were excluded from further analyses because their performances on the second attempt could be due to their superior divergent thinking abilities rather than any effect of the incubation period as mentioned. An additional eight participants were excluded due to failure to follow instructions for the interpolated tasks (N = 6) or UUT (N = 2). Among the remaining 90 participants, 29 were in the MW-prone group (13 male participants; mean age = 21.00 years, SD = 1.63 years), 30 were in the MF-induced group (12 male participants; mean age = 20.80 years, SD = 1.94 years), and 31 were in the control group (13 male participants; mean age = 21.16 years, SD = 1.70 years). No significant differences in age, sex, or the total score of mindfulness was observed across groups (age: *F*(2, 87) = 0.32, *p* = .725, $\eta_{p}^{2}$ = .01; sex: *χ*^2^(2) = 0.14, *p* = .931; the total score of mindfulness: *F*(2, 87) = 0.38, *p* = .686, $\eta_{p}^{2}$ = .01). In addition, both incubation groups rarely thought of the UUT problems during incubation, and we found no group difference in that rating (MW: *M* = 1.14, *SE* = 0.07; MF: *M* = 1.23, *SE* = 0.09; *t*(57) = −0.84; *p* = .404, mean difference = −0.10, 95% CI = [–0.32, 0.13], *d* = 0.21).

### Differences in mind wandering between conditions

As expected, participants’ propensity for mind wandering and diversity of mind wandering differed between the two incubation groups. Specifically, significantly more probe-caught mind wandering occurred under the MW-prone condition (*M =* 4.34, *SE* = 0.31) compared to under the MF-induced condition (*M* = 2.87, *SE* = 0.20) during incubation (*t*(57) = 4.05, *p* < .001, mean difference = 1.48, 95% CI = [0.75, 2.21], *d* = 0.98). By contrast, the MW-prone group scored lower on diversity of mind wandering (*M* = 5.81, *SE* = 0.48) compared to the MF-induced group (*M* = 6.98, *SE* = 0.50), *t*(55) = –1.67, *p* = .100, mean difference = –1.17, 95% CI = [–2.57, 0.23], *d* = 0.45) after excluding two participants from the MW-prone group who experienced difficulty judging the diversity of their mind wandering. That is, participants who engaged in the mindfulness task generated fewer but more diverse thoughts compared to those who engaged in the low-load cognitive task.

The proportions of aware/intentional mind wandering for two participants in the MW-prone group could not be gauged because no mind wandering was caught during incubation. After excluding them, the results showed that the MW-prone group had a higher proportion of aware mind wandering (*M* = 0.93, *SE* = 0.04) compared to the MF-induced group (*M* = 0.77, *SE* = 0.05; *t*(55) = 2.25, *p* = .029, mean difference = 0.15, 95% CI = [0.02, 0.28], *d* = 0.61). Moreover, we observed no group difference in the proportion of intentional mind wandering (MW: *M* = 0.37, *SE* = 0.07; MF: *M* = 0.28, *SE* = 0.06; *t*(55) = 0.98, *p* = .331).

### Incubation effect

Three one-way analyses of variance with condition (MW-prone vs. MF-induced vs. control) as the between-participant factor were conducted to examine the incubation effects on the three indices of divergent creativity. The two-tailed *t-*tests were conducted with the *p* value adjusted using the Bonferroni correction for additional pairwise comparisons.

As mentioned previously, following Kudesia et al. [17] and Yamaoka and Yukawa [26], improvements in the three indices of creativity (i.e., fluency, flexibility, and originality) were defined, respectively, as the additional number of unrepeated responses, categories, and infrequent responses each participant generated during the second attempt compared with the initial trial. An incubation effect occurred if any of the improvements identified in the two incubation conditions surpassed the control condition.

Each participant’s performance was represented as averaged z-scores on the two UUT problems for each of the creativity indices. After excluding unidentified and incomprehensible responses, 2,525 responses remained for bricks and 2,650 for chopsticks. We derived a hierarchical category framework based on these responses and the authors’ guidebook for the task [89] (28 categories for bricks and 24 for chopsticks). Two independent, nonexpert raters blinded to the conditions then classified the participants’ answers into the corresponding categories under this classification framework for each problem. The interrater reliability was high (*K*_*c*_ = .96 and .95 for the chopsticks and the bricks problems, respectively). Discrepancies in classification were resolved by discussion among the raters and the first author. Both raters were male undergraduates from the same university as the participants and recruited specifically for the classification work.

**Fig 2:**
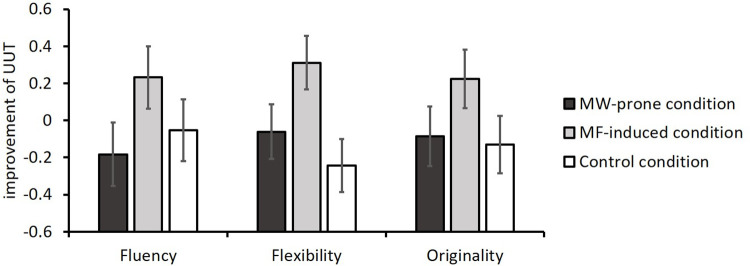
The three aspects of improvement of UUT for participants in the MW-prone condition (N = 29), MF-induced condition (N = 30), and control condition (N = 31).

No group difference was found in fluency, flexibility, or originality during the first attempt (fluency: *F*(2, 87) = 0.07, *p* = .935, $\eta_{p}^{2}$ = .002; flexibility: *F*(2, 87) = 0.24, *p* = .746, $\eta_{p}^{2}$ = .01; originality: *F*(2, 87) = 0.49, *p* = .615, $\eta_{p}^{2}$ = .01). As can be seen in Fig 2, for the improvement of flexibility, a significant main effect of condition was observed, *F*(2, 87) = 3.85, *p* = .025, $\eta_{p}^{2}$ = .08. Further pairwise comparisons revealed that participants under the MF-induced condition generated more categories that were new (*M* = 0.31, *SE* = 0.15) than did the control group (*M* = −0.24, *SE* = 0.14), *t*(87) = 2.73, *p* = .023, mean difference = 0.56, 95% CI = [0.19, 1.21], *d* = 0.70. This result indicates that a significant incubation effect could be brought up through engaging the mindfulness-induced task during the incubation period. However, we observed no difference between the MW-prone group (*M* = −0.06, *SE* = 0.15) and the control group (*t*(87) = 0.89, *p* = 1.000, mean difference = 0.18, 95% CI = [−0.29, 0.74], *d* = 0.23) or between the two incubation groups (*t*(87) = 1.80, *p* = .224, mean difference = 0.37, 95% CI = [−0.05, 0.99], *d* = 0.47).

As for the improvement of fluency and originality, we found no significant main effect of the condition (fluency: *F*(2, 87) = 1.55, *p* = .219, $\eta_{p}^{2}$ = .03; originality: *F*(2, 87) = 1.51, *p* = .226, $\eta_{p}^{2}$ = .03), indicating that no incubation effect for neither incubation groups was observed.

#### Preliminary exploration of the moderating effects of trait mindfulness on the incubation effects

Research has shown that participants with high trait mindfulness could follow mindfulness instructions more readily than those with low mindfulness could [92, 93]. Would it be possible that participants with high mindfulness better benefit from the MF-induced task and showed significant improvement on the second attempt, particularly in fluency and originality on which no incubation effect was found? In other words, whether there exists a possible moderating effect of trait mindfulness on the incubation effect? To explore such possibility, we conducted general linear models for each index of divergent creativity. Condition (MW-prone vs. MF-induced vs. control), mindfulness (the total score of FFMQ), and the interaction between condition and mindfulness were used as predictors in the models. The total scores of FFMQ were centered by mean. Because our sample size was not enough for conducting those models with sufficient power when trait mindfulness was treated as a continuous variable, we reported them as preliminary results. In addition, the post-hoc power for each model of the improvement in UUT were reported.

The general linear model of the improvement in fluency was marginally significant, *F*(5, 84) = 2.17, *p* = .065, $\eta_{p}^{2}$ = .114, *power* = .72. As we expected, significant interaction was observed between condition and mindfulness, *F*(2, 84) = 3.28, *p* = .042, $\eta_{p}^{2}$ = .07. The effect of condition was only significant in participants with FFMQ scores equal to or higher than 1 SD, *F*(2, 84) = 4.89, *p* = .01. Pairwise comparisons revealed that participants with high mindfulness under the MF-induced condition generated more new uses than did those under the control condition, *B* = 0.81, *SE* = 0.36, 95% CI = [0.10, 1.53], *t*(84) = 2.26, *p* = .026, and those under the MW-prone condition, *B* = 0.95, *SE* = 0.32, 95% CI = [0.31, 1.59], *t*(84) = 2.94, *p* = .004. By contrast, participants with high mindfulness under the MW-prone condition did not surpass those under the control condition in fluency, *B* = −0.13, *SE* = 0.36, 95% CI = [−0.85, 0.58], *t*(84) = −0.37, *p* = .714. Both the main effect of condition or mindfulness was not significant (condition: *F*(2, 84) = 1.95, *p* = .148, $\eta_{p}^{2}$ = .04; mindfulness: *F*(1, 84) = 0.05, *p* = .831, $\eta_{p}^{2}$ = .001).

The general linear model of the improvement in flexibility was marginally significant, *F*(5, 84) = 1.90, *p* = .103, $\eta_{p}^{2}$ = .102, *power* = .66. No significant moderating effect of mindfulness was found, *F*(2, 84) = 0.44, *p* = .643, $\eta_{p}^{2}$ = .01. No main effect for mindfulness, either, *F*(1, 84) = 0.35, *p* = .556, $\eta_{p}^{2}$ = .004. Similar to the result of ANOVA, a significant effect was observed for condition, *F*(2, 84) = 4.05, *p* = .021, $\eta_{p}^{2}$ = .09. Further analysis revealed that, participants under the MF-induced condition generated more new categories than did the control group (*B* = 0.57, *SE* = 0.21, 95% CI = [0.17, 0.98], *t*(84) = 2.79, *p* = .007) and those under the MW-prone condition (*B* = 0.39, *SE* = 0.21, 95% CI = [−0.02, 0.81], *t*(84) = 1.89, *p* = .062). No difference was observed between the MW-prone group and the control group (*B* = 0.18, *SE* = 0.21, 95% CI = [−0.23, 0.59], *t*(84) = 0.87, *p* = .389).

The general linear model of the improvement in originality was not significant, *F*(5, 84) = 1.41, *p* = .227, $\eta_{p}^{2}$ = .078, *power* = .51. No interaction or main effect was found (condition: *F*(2, 84) = 1.69, *p* = .190, $\eta_{p}^{2}$ = .04; mindfulness: *F*(1, 84) = 0.02, *p* = .879, $\eta_{p}^{2}$ = .000; interaction: *F*(2, 84) = 1.98, *p* = .145, $\eta_{p}^{2}$ = .05).

### Did any aspect of mind wandering predict the improvement in creativity?

To examine whether the diversity of mind wandering, the number of probe-caught mind-wandering incidents, or the proportions of aware and intentional mind wandering would predict the improvement in fluency, flexibility, or originality, two incubation groups were further combined. Then, we conducted three stepwise multiple regression analyses. Three MW-prone group participants were excluded from those analyses due to having difficulty judging the diversity of their mind wandering or having no probe-caught mind wandering. Variance inflation factors showed that all three models were exempt from multicollinearity (variance inflation factors < 10).

The regression model for flexibility was significant, *F*(1, 54) = 4.54, *p* = .038, adjusted *R*^*2*^ = .06. As predicted, the diversity of mind wandering was the only significant predictor included in this regression model, *B* = 0.09, *SE* = 0.04, 95% CI = [0.01, 0.17], *t*(54) = 2.13, *p* = .038. That is, the higher the diversity of mind wandering was, the more categories that were new were generated after incubation.

In addition, the regression model for originality was significant, *F*(1, 54) = 5.20, *p* = .027, adjusted *R*^*2*^ = .07. Unlike flexibility, the proportion of intentional mind wandering was the only significant predictor in this model, *B* = 0.91, *SE* = 0.40, 95% CI = [0.11, 1.71], *t*(54) = 2.28, *p* = .027. That is, the higher proportion of intentional mind wandering was associated with more ideas that were new and infrequent after incubation.

For fluency, all four predictors were excluded from the regression model, indicating that no aspect of mind wandering significantly predicted the improvement of fluency.

## Discussion

To clarify the inconsistent relationship between propensity for mind wandering and the incubation effect of divergent creativity, we compared the incubation effects brought up by MW-prone and MF-induced states and examined how other aspects of mind wandering during incubation, particularly the diversity of mind wandering we newly proposed, can predict the incubation effect. There are three main findings. First, as predicted, engaging in an MF-induced task was more beneficial for incubation than engaging in an MW-prone task was. Specifically, the MF-induced group, which generated fewer but more diverse mind-wandering incidents than did the MW-prone group during incubation, outperformed the control group in UUT in terms of the improvement in flexibility on the second attempt. No incubation effect was observed for the MW-prone group in any index of UUT. Second, the diversity of mind wandering, but not other aspects, positively predicted the magnitude of the incubation effect regarding flexibility of UUT when we combined the two incubation groups. Third, the proportion of intentional mind wandering during incubation could positively predict the improvement in originality of UUT on the second attempt when we combined the two incubation groups. By contrast, the level of awareness of mind wandering during incubation did not predict any of the incubation effects.

Our study demonstrated for the first time that MF-induced practice as an incubation task was superior to an MW-prone task in eliciting an incubation effect on divergent creativity with respect to flexibility in a head-to-head comparison. Our findings further imply that the advantage of the MF-induced task could be partly due to the increased diversity of mind wandering. This is because improvement in flexibility was only predicted by the diversity of mind wandering, and the MF-induced group had few but relatively diverse mind-wandering incidents, compared with the MW-prone group.

By measuring the diversity aspect of mind wandering during incubation, we also provide a piece of direct evidence supporting for the “free from potent thought” explanation pertaining to the conducive effect of mindfulness induction on incubation that Kudesia et al. [17] proposed. In addition, our results not only replicated their finding that being mindful during incubation facilitated the generation of far-reaching ideas, but also generalized it with a different mindfulness skill. That is, we demonstrated that, for the first time, a specific type of focused-attention meditation skill was as effective as the open-monitoring one in facilitating the incubation effect on a divergent thinking task. Note that some previous studies showed that only practicing the open-monitoring skill, but not the focused-attention one, benefited performance of idea generation or divergent thinking [60, 94]. Regarding the discrepancy, we conjecture that some instructions of focused-attention skill, such as breath counting, might involve more controlled or constrained process than the type we adopted. More studies are required to further clarify which mindfulness skills could benefit creativity and why.

Note that although we examined the effectiveness of mindfulness induction on creativity from the perspective of mind wandering during incubation, we do not mean that this is the only route through which mindfulness induction could benefit creativity. For example, improved affect state, which is often achieved during mindfulness practices [95], is known to be correlated with divergent thinking as well [96].

This study also adds another piece of evidence contrary to the mind wandering account. Similar to what Steindorf et al. [29] and Murray et al. [30] found, our participants in the MW-prone condition did not demonstrate any incubation effect. Instead, our findings suggested that the effectiveness of engaging in a low-load interpolated task might depend on how well participants could let their minds wander freely (for flexibility) or intentionally (for originality). The positive correlation found between the diversity of mind wandering and improvement in flexibility in the MW-prone condition (*r*(27) = .40, *p* = .038) offers a preliminarily support for our conjecture. More studies that assess aspects of mind wandering other than propensity is needed to clarify this issue.

Further, in line with Agnoli et al. [37], who found that daily tendency toward intentional mind wandering was correlated with performance on originality of UUT, we also revealed that intentional mind wandering during incubation is helpful for breaking through mental impasses by generating ideas that are more original. Note that this correlation can be interpreted in the other way around, that is, creative people tend to engage intentionally in mind wandering as some researchers suggested [13, 28, 36]. However, this is not likely to be the case here because we did not find any correlation between the proportion of intentional mind wandering and performance on creativity on the first attempt (*p*s > .23). The finding that the flexibility and originality scores were independently predicted by different aspects of mind wandering also indicates that being capable of avoiding immersion into potent thoughts, and thus having high diversity of mind wandering, might represent a better chance of generating dissimilar or far-reaching ideas relative to an individual’s old ideas, but not necessarily generating ideas that are more unique that few people could consider.

Also note that neither incubation task elicited a higher proportion of intentional mind wandering than the other did. We thus preliminarily explored whether trait-like mindfulness was associated with the proportion of intentional mind wandering across two incubation groups, and no correlation was found (*p* > .99). Several studies have found or suggested that some cognitive styles (such as tendency of self-reflection, non-reactivity to inner experience) or emotional states (such as being anxious or stressed) were associated with trait-like intentional mind wandering [32, 97, 98]. Whether the tendency to engage in intentional mind wandering during incubation would also relate to these individual difference factors deserves further investigation in the future.

Unlike Kudesia et al. [17], we did not find that the MF-induced task benefitted the incubation effect in fluency. Remember that larger incubation effect of fluency was shown for “combined” mindfulness and low-load groups compared to the high-load group in their study. However, there is still no such effect when we did the same analysis (*p* > .71). Again, we explored if participants with high trait mindfulness would better benefit from the MF-induced task because they could follow mindfulness instructions more readily than those with low mindfulness could [92, 93]. As our preliminarily analyses showed, participants with high trait mindfulness under the MF-induced condition did show a significantly larger improvement in fluency, but not in flexibility or originality, compared to the MW-prone and control groups. The data so far indicates that the incubation effect of mindfulness induction on fluency was not as general as that on flexibility and was not through the same route as improving flexibility, i.e., by increasing diversity of mind wandering. More studies with larger sample sizes are required for further investigation on the boundary conditions for the effect of mindfulness induction as an incubation task.

Some limitations of our study and further directions for study are discussed below. First, note that we measured the diversity of mind wandering retrospectively, which was inevitably influenced by the participants’ abilities of memory and meta-awareness. Developing other assessments in parallel with retrospective ratings is thus needed to further strengthen the evidence. Second, whether our findings about the diversity of mind wandering can be generalized to convergent types of creative tasks, which involve idea evaluation exerting on constrained process, remains to be investigated. Finally, although our main findings indicate a mediating role of the diversity aspect of mind wandering on the relationship between the two mental states and the incubation effect, the possible causal relationship among the type of incubation task, the aspect of mind wandering, and the enhancement of creativity remains to be established.

## Supporting information

S1 Dataset Search data for this study. (XLSX)
